# The Prospect of Market-Driven Improvements in Animal Welfare: Lessons from the Case of Grass Milk in Denmark

**DOI:** 10.3390/ani3020499

**Published:** 2013-06-04

**Authors:** Lennart R. Heerwagen, Tove Christensen, Peter Sandøe

**Affiliations:** Department of Food and Resource Economics, University of Copenhagen, Rolighedsvej 25, DK-1958 Frederiksberg, Denmark; E-Mails: tove@ifro.ku.dk (T.C.); pes@sund.ku.dk (P.S.)

**Keywords:** animal welfare, consumption, dairy cows, food policy, grazing, milk

## Abstract

**Simple Summary:**

Increased consumption of animal welfare-friendly products is suggested as one way of addressing public worries about the welfare of farm animals. However, the factors that drive and limit markets for animal welfare-friendly products are poorly understood. Based on an analysis of market for grass milk in Denmark, we conclude that successful cases of market-driven improvements in animal welfare require the joint presence of a number of positive drivers as well as low consumption barriers.

**Abstract:**

Citizens in many European countries urge that the welfare of farm animals should be improved. Policy-makers propose that this could, at least to some extent, be achieved through increased consumption of animal products produced under labeling schemes guaranteeing higher standards of animal welfare. Yet considerable uncertainties exist about the ability of the market to promote animal welfare. So far the consumption of most welfare-friendly products has been limited, and the impact of driving and limiting factors is poorly understood. Reviewing market studies, we identify the factors that have shaped the relatively successful market for grass milk in Denmark. We conclude that the positive drivers such as an appealing animal welfare attribute and animal welfare being bundled with other qualities are essentially the same as those operating in connection with less successful animal welfare-friendly products. It is therefore to be expected that other animal welfare-friendly food products marketed via “natural behaviors” in the farm animals will catch the interest of consumers. However, grass milk consumption has been supported by proper labeling, ready availability and low price premiums as well as multifaceted public support. This suggests that successful cases require the joint presence of a number of positive drivers as well as low consumption barriers.

## 1. Introduction

During the past few decades, livestock production has undergone an intensification in industrialized countries, which has resulted in increased effectiveness but also public concern about the welfare of farm animals. Political decision-makers in the western hemisphere are therefore under on-going pressure to address animal welfare issues in farming [1,2]. However, as improvements into farm animal welfare are often (though not always) linked to increased production costs and/or reduced productivity, a tightening of animal welfare legislation at national level might cause livestock production to be relocated to countries where the standards are lower. Moreover, a policy of restricting imports of livestock products by insisting on domestic animal welfare standards risks infringing international trade agreements [3]. As a consequence, policy-makers have shown a growing interest in strategies where consumer demand for animal welfare-friendly products is expected to drive up animal welfare standards [4,5,6,7,8]. Such strategies rely on the food supply chain actors being able to take advantage of the demand for those types of improved animal welfare standards where the price premiums that certain consumer segments are willing to pay can cover increased productions costs and/or reduced productivity. Thereby, such strategies are not believed to impose financial burdens on producers nor need they disturb free trade agreements. 

A number of factors limiting the market for animal welfare-friendly products have been identified. A study by Grunert *et al*. [9] clearly indicated that animal welfare competes with a long range of other, possibly more important, quality traits, such as taste, tenderness, cut, and safety, in guiding consumer choice. Moreover, even if consumers have a preference for animal welfare-friendly food, not only insufficient labeling but also poor availability and price premiums that exceed their willingness to pay may prevent them from expressing this in their market behavior [10,11]. The possible presence of such consumption barriers imply that the extent to which consumer demand for welfare-approved foods may drive up animal welfare standards remains uncertain.

The purpose of this paper is to explore the prospect of market-driven improvements in animal welfare by considering the Danish consumption of fresh milk from dairy cows with access to grass pastures (which we will refer to as “grass milk”). On the whole people view dairy cows’ access to grass as a form of natural living, which they take to be an important element of animal welfare [12]. So there seems to be little reason to doubt that most people, when they buy grass milk, assume they are contributing to better animal welfare. 

We think that grass milk consumption in Denmark is interesting as a strong case of market-driven advances in animal welfare. About half of Danish sales of fresh milk involve products participating in labeling schemes which guarantee that the cows are kept on grass. This market share subdivides into organic milk (about 29% in 2011, according to Organic Denmark [13]) and the private labeling scheme “Lærkevang^®^” (about 20% in 2011 and 2012 [14]). Organic milk is the most important organic livestock product in Denmark closely followed by organic eggs with a market share of 22.5%. In comparison, organic beef and pork have about 3% and 1.5% of the total market respectively (Organic Denmark [13]). At the European level, the 50% market share of grass milk seems to be matched only by animal welfare-friendly eggs in some countries [15]. 

Grass milk consumption should therefore provide a good case study of the ability of markets to promote animal welfare. Hence, it should help to understand both the challenges and opportunities of market-driven management of animal welfare to different forms of livestock production.

The paper is organized as follows. First, we briefly introduce the development in the use of grazing in Danish dairy production and how grazing may affect animal welfare. Second, we analyze some of the factors which seem to drive the market for grass milk in Denmark. Third, the prospect of generalizing the lessons learned from developments in grass milk consumption is discussed.

## 2. Grazing of Dairy Cows

In Denmark, and in many other countries in North-West Europe, dairy production has shifted dramatically over the last 30 years towards fewer and larger farms with higher productivity per cow [16]. According to a 2009 report on dairy cow husbandry, the number of cows in Denmark halved during this period to about 500,000, yet the production volume remained relatively stable. This development is explained by the rise in average annual milk yield, which went from 5,200 kg in 1980 to 9,000 kg in 2006. The report further explains that radical growth in average farm size, up from 25 cows per farm in 1980 to 106 cows in 2006, is expected to continue, reaching 193 cows per herd by 2015 [17]. 

The increasing size of dairy herds has had a significant impact on grazing routines. Cows are now more likely to be stabled on an all-year basis owing to insufficient pasture and practical difficulties in moving, feeding and milking the animals in pasture systems. According to Kristensen [18], it is expected that 15% of conventional dairy cows in Denmark are put to grass in 2015, as compared with 67% in 2003. In the future the number of dairy cows kept on grass may therefore largely depend on consumer demand for grass milk and other dairy products guaranteeing grazing under labeling schemes. 

It is important to stress that there are different opinions on how grazing affects the welfare of dairy cows. It appears from a 2006 investigation commissioned by the Danish government that grazing allows dairy cows relatively free movement, natural rest, and freedom to run and walk; and that in these respects it can be regarded as a benefit for the animals. This argument is supported by studies showing that cows on pasture tend to get more exercise than cows kept indoors, have longer rest periods, and experience lower level of aggression (as weaker animals are able to escape dominant ones). On the other hand, it also appeared that the health benefits of grazing are more ambiguous. Grazing cows may have lower frequency of the hoof disease interdigital dermatitis (presumably, because pastures keep hooves in a cleaner state than stable floors), but also higher frequency of other hoof diseases, such as interdigital phlegmon (possibly as a result of stony and muddy driveways). Similarly mixed pictures emerge when metabolic disorders are examined, with some appearing to be more widespread among grazing cows and others less. An increased risk of parasite infections is also found among grazing cows [17]. 

## 3. Grass Milk Consumption

### 3.1. Micro and Macro Factors Influencing Grass Milk Consumption

Thøgersen [19] distinguishes between micro and macro factors influencing food consumption. Micro factors are related to individual consumer behavior and include the motivation that drives consumers towards particular food choices but also consumption barriers such as limited availability, poor labeling and high price premiums. Our analytical focus is initially on these micro-factors. Yet Thøgersen recommends that analyses of food consumption should also encompass macro factors which frame the individual consumer choice. Therefore we also focus on governmental support of organic production and some aspects of the pricing of organic food. 

### 3.2. Grass Milk Varieties on the Danish Market

The main varieties of grass milk on the Danish market are organic milk and Lærkevang. Organic milk appeared in mainstream Danish supermarkets as early as 1988, as the result of cooperation between the largest Danish retail chain FDB and several dairy companies [20]. Organic production rules require dairy farmers to keep dairy cows on grass for at least six hours daily during the grazing period, which stretches from the 15 April to 1 November—if weather conditions and the state of the animals allow it. A number of organic requirements also relate to the quality and condition of grazing areas. Also, organic certification comes with rules on daily exercise, the treatment of calves, the provision of daylight, and space for stalled animals [21].

Lærkevang (meaning “Lark Meadow”) was introduced later, in 2007, by the main dairy producer in Denmark, Arla Foods. Lærkevang was marketed as a quality milk product guaranteeing fresh milk from cows that are allowed on pasture. The Lærkevang labeling scheme guaranteed that dairy cows were kept on grass for four successive months during the summer for at least six hours daily. Dairy farmers operating under the labeling scheme were also required to provide their cows with a certain quantity of grass during the winter season [22]. In other respects, Lærkevang farms were subject to general legislation imposing minimum requirements on animal welfare. Lærkevang is the direct descendant of “Arla Ekspres”, a quality label under which Arla had marketed milk in terms of its freshness, guaranteeing delivery to retailers within 24 hours [23]. Difficulties in branding Lærkevang as being a non-organic quality milk from cows on pasture together with a marketing study showing strong preference among Danes for fresh milk, led Arla to change their marketing of Lærkevang in 2013 back to being fresh milk delivered to retailers within 24 hours. The guarantee that cows are allowed on pasture was abandoned [24]. The development in the Lærkevang brand clearly indicates that animal welfare is perceived as a positive but not necessarily strong attribute that is difficult to brand on its own. The impact of the latest change in marketing strategy on Lærkevang will be interesting to follow.

We refer mainly to organic milk, because empirical data on consumer perceptions and behavior are available for it, but not for other types of grass milk. This is a limitation of our analysis. However, we also draw on studies which shed light on consumer attitudes to grazing as an attribute of milk. This allows us to extend the analysis across the wider field of grass milk consumption.

### 3.3. Grazing Cows as a Driving Factor

A range of motives have been found to drive organic milk consumption in Denmark. In a survey reported by the Danish Consumers’ Cooperative Society (FDB) [25], human health was found to be the most significant stated motive; this was followed by animal welfare, better taste/better quality and environmental concerns. In a study combining purchase data and information on consumer attitudes to organic food, Andersen [26] also identified concerns about human health as a more important factor for the choice of organic milk than environmental concerns. Grunert *et al*. [27] investigated attitudes to organic dairy products and found that among Danish consumers there was a widespread belief that organic dairy production is better for animal welfare, better for the environment, and (albeit to a lesser extent) offers better working conditions for producers. Finally, the significance of better taste/better quality is emphasized in Andersen [28], which reported that about 40% of participating respondents experienced a better taste when drinking organic instead of conventional milk. In a significant number of publications, concerns about human health, animal welfare and the environment have all been described as drivers of the consumption of animal welfare-friendly foods in general, e.g., [29,30,31].

Some studies indicate that the health of young children provides a particular motive for purchasing organic milk. In FDB [25] human health was found to be an especially strong motive for purchasing organic milk in families with young children. Child health has also been identified as a reason for switching to organic milk in qualitative studies conducted in Denmark [32] and the UK [33]. Other studies, such as Andersen [26], could not confirm the relationship, however. Although the current evidence appears to be somewhat mixed, the role of milk in some cultures lends plausibility to the idea that children’s health is a specific driving factor here. An ethnographic study conducted in ten European countries, including Denmark, showed that milk was associated with family, home and the parental care of children. Indeed milk was found to have a strong moral meaning as “*the* drink that good parents provide for their children” [34]. It can be seen, then, that to the extent that milk is regarded as part and parcel of childcare, parents may have a particularly strong motive for purchasing milk products which they perceive as healthy.

The cow’s opportunity to graze seems to have some importance in the Danish consumer’s thinking about milk. Denver and Christensen [35], asked participants in their study to categorize a selection of milk attributes as very positive, somewhat positive, negative, or of no importance. Next to the absence of pesticide and medicine residues, the respondents deemed grazing the most important positive attribute, with about 60% of them assigning a high degree of importance to grazing. Consumers also assigned levels of importance to the milk not being close to expiration date, organic labeling, organic fodder, and environment-friendly cartons. It emerged that grazing was valued by Danish consumers, but it was also clear that organic milk, and other types of grass milk, possess other attributes which attract consumers. Thus, even though we have chosen to focus on the grazing aspect of organic and Lærkevang milk, some purchases of these products may be only partly, or in some cases not at all, due to this attribute. 

The question why grazing is thought to be valuable by many consumers is under-researched, but it seems very likely that animal welfare is a vital part of the reason. Boogaard *et al*. [36] showed that Dutch citizens who visited dairy farms believed it to be important that cows are able to live in a “natural” manner. This included being allowed to graze. Many citizens also believed that the grazing of dairy cows is an important aspect of landscape aesthetics and rural culture. This is in keeping with the finding, reported in Oudshoorn *et al*. [37], that Danish and Dutch farmers assign decisive importance to grazing as the “image” of organic dairy and a representation of its “naturalness”. Studies of public attitudes to farm animals in Denmark and other European countries generally find that people think of the freedom to perform natural behaviors as a vital part of animal welfare [12,38]. 

Grazing is also connected with purchasing motives involving human health. Denver and Christensen [35] confronted 900 consumers with the following statement “Milk from a cow that has been out in the pastures and eaten fresh grass contains more vitamins and more healthy fatty acids. Were you aware of that?” As many as 77% replied, that they were aware of this relationship. Even though eagerness to appear knowledgeable introduces potential bias here, the result still indicates a positive attitude to the healthiness of grass milk. 

To sum up, we are not able to link grazing with a single value such as animal welfare but with a whole range of values, some of which are linked to consumer health. Furthermore, it seems fair to conclude that similar bundles of values are associated with organic and non-organic grazing cows and that the grazing requirement is an important part of the value of organic milk. 

### 3.4. Low Consumption Barriers as a Driving Factor

Organic milk is available in most supermarkets and even in discount stores. Wier *et al*. [39] found that 95% of organic milk in Denmark was sold through these retail channels. Also, organic milk is marketed by several dairy companies who also sell non-organic milk and is generally available in all popular types, including skimmed, mini, semi-skimmed, whole and buttermilk. Lærkevang can also be purchased in most popular types [40]. The relatively easy availability of organic milk and Lærkevang in Denmark is an important factor in the success of grass milk; consumers in other countries find it difficult, or too time-consuming, to locate animal welfare-friendly products [10]. So, although lack of availability might be a major barrier to animal welfare-friendly consumption in general, it does not seem to be an obstacle to grass milk sales in Denmark. 

In Denmark obstacles to organic consumption caused by poor or untrustworthy labeling have been significantly reduced by the national organic label (the so-called “Ø-label”) which was introduced in 1989 and has guaranteed governmental control of the certification of production, processing, packaging and labeling of organic food products since then. The significance of the Ø-label in Denmark was emphasized in a survey of trust showing that more than 80% of Danish consumers believed that products marketed as organic under the Ø-label actually were organically produced in most cases [41]. No studies of the trustworthiness of the label Lærkevang have been found.

The Ø-label is only one example of governmental support of organic agriculture in Denmark. This support has been regulatory, financial and advisory, and it has helped to establish and sustain a stable and standardized source of supply. Demand-side tools, including not only labeling but also consumer information and marketing, have also been exploited [42]. Recently, two studies drawing on international data from Denmark, Sweden, the UK, and the USA have shown the significance of governmental support. In the first study, Daugbjerg and Sønderskov [43] examined the effect of governmental support in the four countries by setting degrees of public support beside the development of the market for organic food. After testing for a number of alternative explanations, they found that the most significant growth in organic markets was likely to occur when the level of governmental intervention was high and demand- and supply-side instruments were combined in an integrated approach—as happened in the Danish case. In the second study, Sønderskov and Daugbjerg [41] investigated whether governmental involvement influenced consumer confidence in organic labeling in the four countries. They concluded that a government’s involvement in organic labeling does seem to increase confidence, as is illustrated in the case of the Danish organic market. 

Finally, an international and categorical comparison of organic price premiums has confirmed that organic milk in Denmark is priced relatively low. Wier *et al*. [39] compared the price premiums of eight organic products in Denmark and five such products in the UK. This revealed that the price premium on organic milk was second lowest among organic products in Denmark (next to yoghurt) and lower than that of any of the five organic products sold in the UK. Another Danish study found that the 23% price premium on organic mini milk was lower than the premiums placed on organic varieties of carrot, potato, egg, pork and beef [44]. Hence, a small price premium may be one of the driving factors of organic milk. As the price of Lærkevang is bounded above by the price of organic milk, the price premium of Lærkevang is also small [14].

Wier and Calverley [45] suggest that price reductions in animal welfare-friendly food might be achievable in many cases, as high price premiums are in part features of immature markets characterized by inefficiency and higher costs of transport, processing and packaging. The premiums may shrink as markets increase, in other words. However, additional costs associated with organic production also shape the price premiums. In their study of the Danish organic livestock production, Hermansen *et al*. [46] argue that organic rules have, in several respects, allowed organic milk production to follow the same structural development as conventional milk production. Although organic rules prescribe grazing and other husbandry requirements, organic dairy production has moved towards fewer, but larger, herds of high-performing animals. Hermansen *et al*. also found that the differences between organic and conventional production are much more significant in the case of pork, eggs and broiler chickens, where they push up production costs and contribute to high price premiums. These observations indicate that even though price premiums might shrink as the markets for animal welfare-friendly products increase they are likely to be bounded from below by increased production costs. 

## 4. Discussion

Danish consumers purchase organic milk because they regard it as healthier, better for the welfare of cows, tasty and better for the environment. The grazing of dairy cows appears as to be a core part of the explanation for this belief, as a narrative about free-roaming animals in natural surroundings is likely to have a general appeal to consumers. It seems to be possible in general to market animal welfare-friendly products through appealing narratives about free-ranging animals. 

Looking across the literature, consumers’ interest in animal welfare seems very much focused on natural behaviors. In a survey documented in FDB [12], Danish consumers were asked to identify the three aspects of animal welfare they felt were most important. The survey confirmed the widespread enthusiasm for allowing farm animals to express natural behaviors: this aspect was found to be the most important one, with support from about 75% of respondents. A low incidence of diseases, no tail docking, and low mortality rates were all chosen by less than 20%, and as such they were the least important aspects of animal welfare. Similarly, a study conducted by Evans and Miele [38], based on 48 focus group interviews in seven European countries, found that consumers in general had limited understanding of so-called “animal-centered welfare issues” (*i.e.*, issues linked to problems which can be seen in reactions of the animals, for example in the form of diseases) and little knowledge of the biology, physiology, behavior, diseases or typical injuries of specific farm animals. Rather, consumers tended to focus on living environments, and on whether these make natural behaviors possible or impossible. This indicates that the interest of consumers in animal welfare is selective, and that aspects of animal welfare of a more technical nature, or directly related to negative stories, such as disease or mortality, are less suited for marketization.

In the case of organic grass-milk, like with some other animal welfare-friendly products, animal welfare is bundled with other attributes under claims of sustainable, including the absence of pesticide and medicine residues, natural, and traditional production methods. It also appears to be of great importance that typical consumption barriers in terms of limited availability, poor labeling and high price premiums are relatively low, in the case with grass milk. However, the cases where consumption barriers can be reduced as significantly as they are in the case of grass milk consumption do seem to be limited. In particular, the low price premiums connected with grass milk may turn out to be rather exceptional.

Finally, multifaceted governmental support, encompassing demand-side as well as supply-side instruments, appears to be a vital factor in the large market share of organic products in Denmark, including the market share of organic milk. If this is right, the potential to develop strong market-driven animal welfare in other countries may depend in a similar way on the willingness of governments in those countries to provide similar support. 

Grass milk consumption suggests that cases where advances in animal welfare are driven principally by the market, or consumer demand, will generally be backed by the joint presence of several strong driving factors and small limiting factors—or, possibly, by one or two individual factors that are extremely strong**.**

Table 1 provides some examples of the potential marketability of various animal welfare-friendly products based on the presence (+) and absence (−) of important drivers of consumption. The case of grass milk consumption is exemplary, and thus the likelihood of successful cases of market-driven improvements in animal welfare increases with the + score. The overview assumes Danish market conditions.

The table illustrates that successful harnessing of the market is feasible and has been the case with grass milk. This conclusion is based on the fact that grass milk has a positive score on all factors. Animal welfare-friendly shell eggs are also doing well on the market. They are characterized by a well-defined EU labeling scheme and a large market share (which makes them readily available). The price premiums are also relatively low—especially in the case of barn eggs [47,48]. The animal welfare narrative is related to space and/or outdoor access, which play a key role in consumer perceptions of good animal welfare. Evans and Miele [38] found that in some cases consumers associated animal welfare-friendly eggs with better taste and/or good appearance (nice looking yokes). However, in general it seems fair to say that “shell eggs”, as product category, is weak on quality attributes, and this of course reduces the significance of this factor. Whereas the animal welfare attribute in both cases have had a strong appeal among consumers, the additional vital driving force in the case of grass milk seems to have been the link between grass-milk and a health perception and possibly low price premiums whereas the additional driving force in the case of free-range eggs is more likely to be the clear labeling of animal welfare-friendly products.

**Table 1:** Examples of the potential marketability of animal welfare-friendly products.

	Appealing animal welfare narrative	Animal welfare bundled with other attractive attributes	Proper labelling	Commonly available	Low price premium
**Grass milk**	+	+	+	+	+
**Organic/free-range /barn shell eggs**	+	−	+	+	+
**Organic/free-range meat products**	+	+	+	−	−
**Pork from 100% free sows (not marketed)**	+	−	+ (Assumed)	+ (Assumed)	+
**Chicken without foot pad dermatitis (not marketed)**	−	−	+ (Assumed)	+ (Assumed)	+

The animal welfare profile of organic and free-range meat also agrees with consumer perceptions of animal welfare, and there is firm evidence that consumers associate organic and free-range production with healthier and better-tasting food products [38]. However, these positive aspects seem to be outweighed by high price premiums and occasional low availability [48].

Pork from 100% loose-housed sows is not a genuine case of market-driven improvement in animal welfare. From January 2013 it is an EU requirement that sows are loose during pregnancy but can be kept in stalls after mating/insemination and while they are farrowing and lactating [49]. However, one can imagine this sort of product being made readily available in supermarkets under private labels. If that were to happen, it seems likely that the price premium would be lower than that for free-range products, given the limited additional production costs involved in the latter, and that many consumers would ideally like to see pigs not being confined at any time. However, loose-housed sows in an indoor environment might not be easily be bundled with other attributes under the wider umbrellas of “naturalness” and “traditional husbandry”. They may therefore lack some of the appeal associated with free-range and organic products. 

The last example in Table 1, chicken without foot pad dermatitis, is another imagined case of market-driven animal welfare. Problems with foot pad dermatitis in Danish broiler production were reduced markedly between 2001 and 2004 when new regulatory instruments were introduced [50]. Yet if chicken without this variety of dermatitis were to be marketed as an animal welfare-friendly product, it is likely that, while the price premium could be kept low, consumers would not regard an improvement focused on reducing a disease appealing; and it may also prove difficult to bundle this selling point with other quality attributes. 

## 5. Conclusion

The purpose of this paper has been to explore the prospects of market-driven improvements in the welfare of animals kept for food production. Danish consumption of grass milk was chosen as a case in which the market for an animal welfare product was developed relatively successfully. The aim was to use this case to identify factors that might be important for demand-driven improvements in animal welfare more generally.

First, the analysis of the Danish market for grass milk revealed that many consumers regard grazing as an attractive milk attribute because they connect it with animal welfare and human health. Moreover, the analysis of the Danish market for organic milk revealed a range of purchasing motives among consumers besides the concern for animal welfare: human health, and perhaps specifically the health of young children; an interest in better taste and/or food quality; and environmental concerns. Secondly, it emerged that the negative impact of commonly found barriers to consumption, such as limited availability, poor labeling and high price premiums, was low. Thirdly, the presence of multifaceted governmental support for organic production appears to have been an important driving factor in the large market share of organic milk.

At a first sight, this may give cause for some optimism about market-driven gains in animal welfare, because the drivers of grass milk consumption do not seem to differ essentially from drivers that could apply to other animal welfare-friendly products: A relatively straightforward narrative about free-roaming animals in natural surroundings supplemented with other attractive attributes was found to be a generally useful formula. However, the low negative impact of consumption barriers seemed to be quite specific to the Danish market and to grass milk just as the importance of governmental support should not be underestimated. 

This suggests that the market can be successfully harnessed to drive improvements in animal welfare only in the presence of a combination of attractive attributes and low consumption barriers. On this basis the prospect of successful cases of market-driven improvements in animal welfare seems rather restricted and conditioned by long-term, extensive governmental support and relatively low production costs. In a somewhat broader perspective, retailers and other large food chain actors are increasingly seen to include animal welfare in their Corporate Social Responsibility strategies [51]. Hence, the prospects of shifting governmental focus from stimulating animal welfare-friendly niche-products to stimulating food chain actors to include animal welfare as part of their branding strategy is worth investigating in the pursuit of improved farm animal welfare.

We expect that the experiences learned from the Danish case of grass milk are useful for assessing the prospects of market-driven animal welfare for a wide range of animal products and a wide range of western communities. We have learned that even in Danish society where the organic consumption is among the highest in the world [52] and citizens state a high level of concern for animal welfare [53], a range of favorable conditions need to be present if animal welfare is to be improved by market initiatives. Hence, market-based animal welfare does not come by itself.
